# A monoclonal antibody raised against bacterially expressed *MPV17* sequences shows peroxisomal, endosomal and lysosomal localisation in U2OS cells

**DOI:** 10.1186/s13104-016-1939-0

**Published:** 2016-02-27

**Authors:** Hans Weiher, Haymo Pircher, Pidder Jansen-Dürr, Silke Hegenbarth, Percy Knolle, Silke Grunau, Miia Vapola, J. Kalervo Hiltunen, Ralf M. Zwacka, Elmon Schmelzer, Kerstin Reumann, Hans Will

**Affiliations:** Heinrich-Pette-Institute, Leibniz-Institute for Experimental Virology, Martinistrasse 52, 20251 Hamburg, Germany; Bonn-Rhein-Sieg University, von Liebig Strasse 20, 53359 Rheinbach, Germany; Institute for Biomedical Aging Research, University of Innsbruck, Rennweg 10, 6020 Innsbruck, Austria; Institutes of Molecular Medicine and Experimental Immunology, Universität Bonn, 53105 Bonn, Germany; Department of Biochemistry, Biocenter Oulu, University of Oulu, FI-90014 Oulu, Finland; School of Biological Sciences, University of Essex, Colchester, CO4 3SQ UK; Max Planck Institute for Plant Breeding Research, Carl-von-Linné-Weg 10, 50829 Cologne, Germany

**Keywords:** MPV17 monoclonal antibody, Mitochondrial DNA depletion syndrome, Lysosomes, Endosomes, Mitochondria, Peroxisomes

## Abstract

Recessive mutations in the *MPV17* gene cause mitochondrial DNA depletion syndrome, a fatal infantile genetic liver disease in humans. Loss of function in mice leads to glomerulosclerosis and sensineural deafness accompanied with mitochondrial DNA depletion. Mutations in the yeast homolog *Sym1*, and in the zebra fish homolog *tra* cause interesting, but not obviously related phenotypes, although the human gene can complement the yeast Sym1 mutation. The MPV17 protein is a hydrophobic membrane protein of 176 amino acids and unknown function. Initially localised in murine peroxisomes, it was later reported to be a mitochondrial inner membrane protein in humans and in yeast. To resolve this contradiction we tested two new mouse monoclonal antibodies directed against the human MPV17 protein in Western blots and immunohistochemistry on human U2OS cells. One of these monoclonal antibodies showed specific reactivity to a protein of 20 kD absent in *MPV17* negative mouse cells. Immunofluorescence studies revealed colocalisation with peroxisomal, endosomal and lysosomal markers, but not with mitochondria. This data reveal a novel connection between a possible peroxisomal/endosomal/lysosomal function and mitochondrial DNA depletion.

## Findings

### Background

Mutations in the human *MPV17* gene have been firstly discovered to be causal for the lethal liver disease Mitochondrial DNA Depletion Syndrome (MDDS) by Spinazzola et al. in 2006 [21]. Since then various additional mutations within this gene were described to cause the syndrome [5, 8, 23]. In contrast to other proteins of which gene mutations cause MDDS such as POLG, TK2, or DGOUK [4], this protein is not obviously involved in replication or in nucleic acid metabolism, but appears to be a membrane protein of unknown molecular function [21, 25, 26]. *MPV17* gene knockout in mice has been described earlier as causal of glomerulosclerosis [2, 14, 25] and inner ear disease [11], reminiscent of chemical damage by Adriamycin [15]. Although these mice also showed mitochondrial DNA depletion, they displayed no major liver phenotype [24]. A functional link between the PRKDC repair protein, the mouse kidney phenotype as well as the mitochondrial depletion phenotype has been established by Papeta et al. [15]. Human MPV17 protein expression can rescue the phenotype in transgenic mice, negative for murine *MPV17* [20]. A functional homolog of the MPV17 protein was identified in yeast (Sacharomyces cerevisiae) and the human gene can rescue the yeast phenotype in *Sym1* negative yeast as well [22]. In addition, a mutant in the zebra fish (Dario rerio) *MPV17* homolog *tra* has been described [12]. The yeast and fish mutant phenotypes appear remarkably different from the mutant phenotypes in mammals: *Sym 1* negative yeast fails to grow at elevated temperature in ethanol [22], while *tra* negative fish are viable but show a transparent appearance [12]. Finally, MPV17 is a member of a protein family, including MPV17 like proteins [6, 19] and the peroxisomal membrane protein PXMP22 [7, 18]. The hypothesis that MPV17 protein might constitute a channel allowing—in vertebrates—nucleotides or—in yeast—metabolic intermediates to pass though internal membranes was recently reviewed [13].

The publication describing the causative role of *MPV17* mutations in the human liver disease MDDS included an analysis of the intracellular localisation of the MPV17 protein in human cells based mainly on studies of transfected cells over-expressing a c-terminally tagged recombinant protein, it was stated that this protein localised to the inner membrane of mitochondria [21]. Supporting this notion, it has been established that the yeast homolog of MPV17, SYM1, indeed localises to this cell compartment [17, 22]. An earlier study [26], however, had localised the *MPV17* gene product to peroxisomes, again based on immunofluorescence data and supported by the similarity of the protein to the bona-fide peroxisomal membrane protein PXMP22 [7].

To clarify this contradiction we here present co-localisation studies using novel anti-human MPV17 monoclonal mouse antibodies.

### Methods

#### Cell lines and tissue culture

Human osteosarcoma cells (U2OS) cells were originally obtain from the American tissue culture collection (ATCC^®^ HTB-96™), kept at the Heinrich Pette Institut for several years and used in [10]. Primary murine embryonic fibroblasts (MEF) were produced from day 11 *MPV17* +/+ and *MPV17* −/− embryos respectively according to Zwacka et al. [26]. U2OS and mouse MEF cells were cultured under standard conditions. Stable transformants were selected using 1 mg/ml G418 (Life Technologies). Hybridomas were generated as described [26], and clones were grown in RMPI media including 10–20 % FCS. The clones were monitored for IgG production by immunoblotting using anti-IgG antibodies and the ECL System (Pierce) for detection. Antibodies were purified using a Protein G Sepharose column (Life Technologies) according to the manufacturer’s instructions.

#### Antibodies and organelle detection

Commercial primary antibodies against MPV17 were: mouse monoclonal anti-human MPV17 antibody (60103-1-Ig, Proteintech), rabbit anti-human MPV17 polyclonal antibody (10310-1-AP, Proteintech); rabbit anti-human MPV17 polyclonal antibody (ab93374, Abcam); goat anti-human MPV17 polyclonal antibody (sc-109551, Santa Cruz), rabbit anti-MPV17 C-terminal region polyclonal antibody ARP73712-P050 Insight Biotechnology), rabbit anti-MPV17 N-terminal region polyclonal antibody AP8749a-ev-AB, BioCat).

Further primary antibodies used were: mouse polyclonal anti-human catalase antibody (Abcam ab88650); mouse monoclonal anti-complex IV mitochondrial subunit I (Invitrogen 459600); rabbit polyclonal anti-human PMP70 antibody (gift from Wilhelm Just, University of Heidelberg); rabbit anti-human Rab 7 antibody (Sigma R4779); goat anti- human cathepsin D antibody (Santa Cruz sc 6486); rabbit anti-human EEA1 antibody (Novus-Biologicals, NB-300-502); goat anti-human LAMP1 antibody (Santa Cruz, sc 8098); mouse monoclonal anti-flag antibody (Sigma, M2, F3165), mouse monoclonal anti-human beta-actin antibody (Sigma, AC74, A5316).

Primary monoclonal Antibodies 5D2 and 6F5 were isolated from mass cultures and purified over protein G Sepharose (Life Technologies). A Rabbit polyclonal anti-human PMP70 antibody was a gift from Wilhelm Just, University of Heidelberg. Further primary antibodies used were: rabbit anti-human Rab 7 antibody (Sigma R4779); goat anti-human cathepsin D antibody (Santa Cruz sc 6486); rabbit anti-human EEA1 antibody (Novus-Biologicals, NB-300-502); goat anti-human LAMP1 antibody (Santa Cruz, sc 8098); mouse monoclonal anti-human beta-actin antibody (Sigma, AC74, A5316).

Secondary antibodies: HRP labeled anti-mouse IgG antibody (Pierce); Alexa Fluor 555 and Alexa Fluor 488 with appropriate anti-mouse, anti-rabbit, and anti-goat specificity were from Life Technologies. For mitochondrial staining, MitoTracker Red 7510 was employed according to the manufacturer’s instructions.

#### Cell extracts and immunoblotting

Cell extracts were prepared and the immunoblotting was performed as described by Kinkley et al. [10] and Pircher et al. [16].

#### Immunofluorescence analysis

**Fig. 1:**
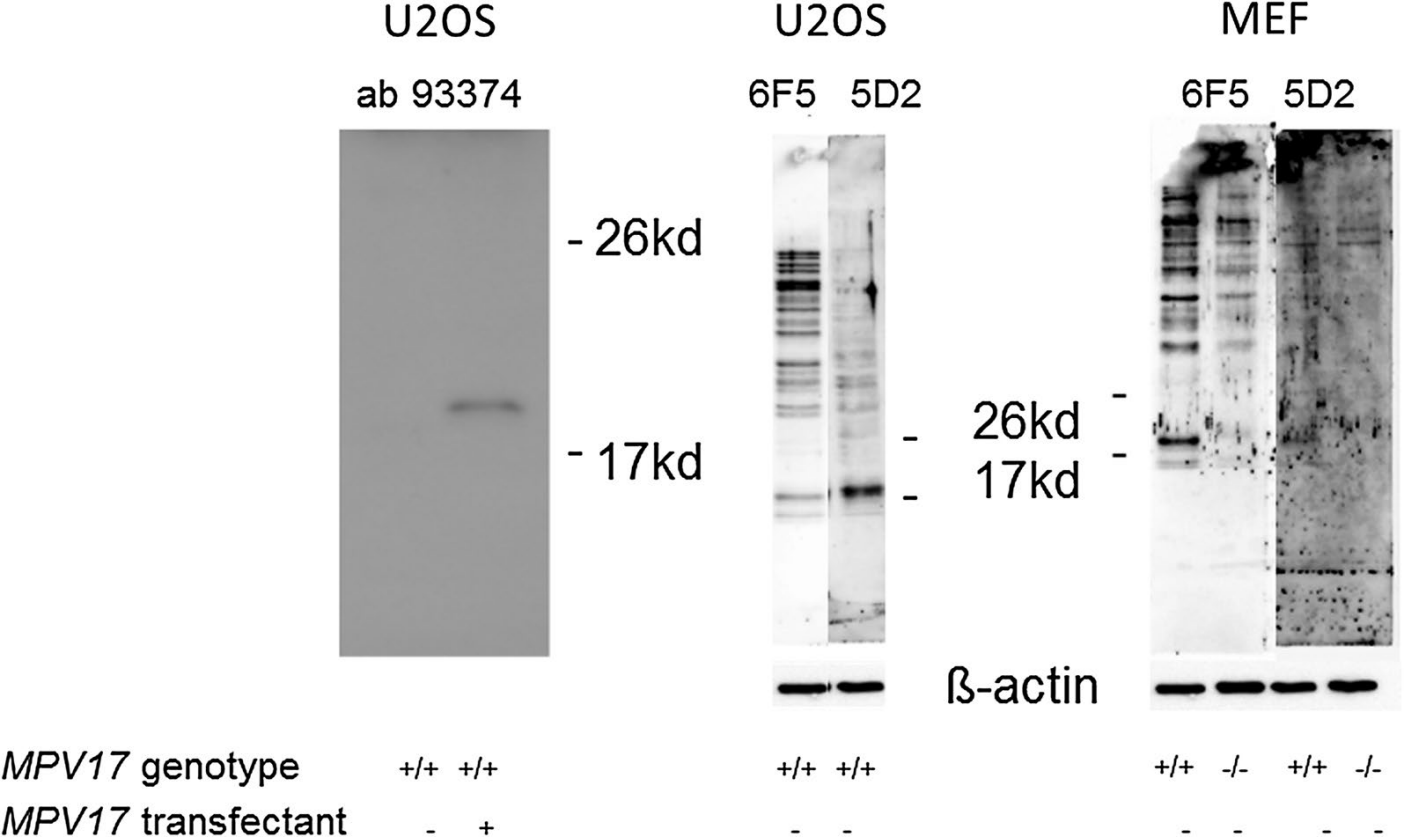
Western blot immunoreactivity of the polyclonal anti-MPV17 antibody Ab 93374 (*left*) and the monoclonal anti- human MPV17 antibodies 6F5 and 5D2 on extracts of human and murine cells of different *MPV17* genotypes. *Left* extracts from human U2OS cells, untransfected or transfected with the *MPV17* expression clone SC118652 (origene), were probed with the antibody ab 93374 (abcam). *Center* untransfected U2OS cells with *MPV17* +/+ endogenous genotype probed with the monoclonal antibodies 6F5 and 5D2. *Right* Murine embryo fibroblast (MEF) cells of *MPV17* +/+ or *MPV17*-/- genotype probed with the monoclonal antibodies 6F5 and 5D2. Indicated marker sizes were transferred from Ponceau stained marker bands run in parallel. Loading was controlled as indicated by reaction of the filters with an anti-ß-actin antibody. 10 μg of protein were separated on SDS gels and analysed by Western blot according to [16] (*left panel*), and Kinkley et al. [10] respectively

Immunofluorescence analysis was performed essentially as described by Kinkley et al. [10]. In brief, cells were grown on cover slips, fixed with 4 % paraformaldehyde in PBS (10 min at room temperature), washed twice with PBS, permeabilized with 0.5 % Triton X-100 in PBS (10 min at room temperature), washed three times in PBS and then blocked for 1 h (at room temperature) or overnight (4 °C) with 3 % BSA in PBS. Cover slips were then incubated in 200 μl of primary antibodies, diluted 1/200 in PBS, for 1 h, washed three times with PBS and then incubated with the appropriate secondary antibodies for 1 h. The cover slips were then washed three more times in PBS and mounted on slides using Mowiol (Calbiochem). The cells were analyzed by indirect immunofluorescence microscopy using a Zeiss LSM confocal microscope or a MicroRadiance confocal scanning system (Bio-Rad) in combination with a Zeiss Axiophot microscope (Fig. 1). Colocalisation analysis was performed with Fiji (Image J).

#### Plasmid constructs

An *MPV17* expression construct (SC118652) was from origene. For details see: www.origene.com/cdna/.

### Results

**Fig. 2:**
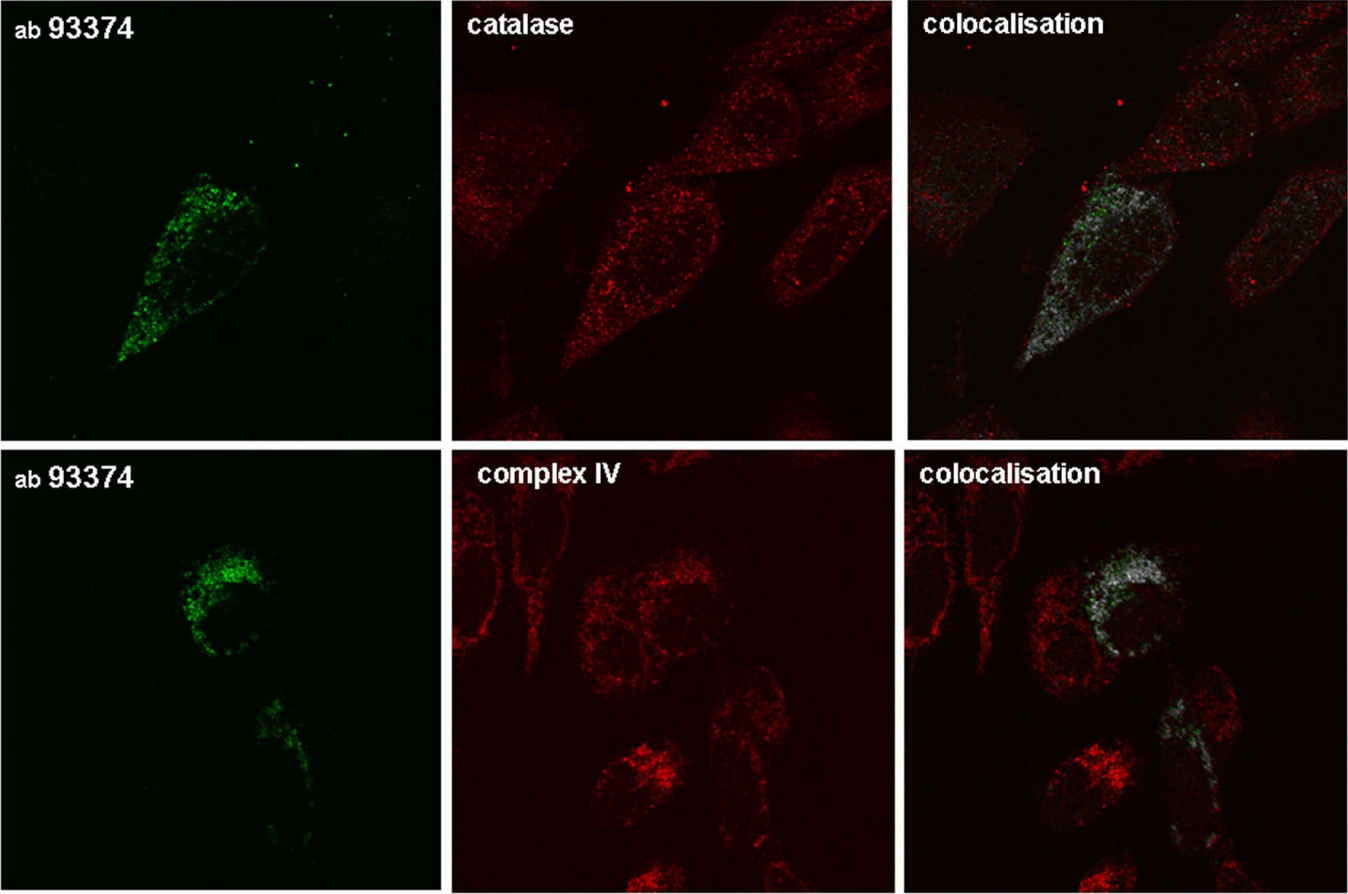
Ab93374 (Abcam) recognizes MPV17 specific structures in U2OS cells transiently transfected with an *MPV17* expression construct in immunofluorescence analysis. Ab93374 signal (*green*) is largely missing in nontransfected cells. The immunoreactivity does colocalise with peroxisomal (anti-catalase, mouse polyclonal, Abcam ab88650) and mitochondrial (anti-complex IV mitochondrial subunit I, mouse monoclonal, Invitrogen 459600) markers (*red*). Immunofluorescence (IF) method was according to Pircher et al. [16] using a MicroRadiance confocal scanning system (Bio-Rad) in combination with a Zeiss Axiophot microscope. Colocalisation analysis was performed using the Fiji software of Image J and is illustrated by *white dots*

Commercially available anti-human MPV17 antibodies from different sources (Abcam ab93374; Proteintech 10310-1-AP, 60310-1-Ig; Santa Cruz Biotechnology SC109551; Insight Biotechnology ARP73712-P050; BioCat AP8749a-ev-AB) were tested in western blot (Fig. 1) and immunofluorescence studies (Fig. 2) on human osteosarcoma cells (U2OS) transfected with a human-*MPV17* expression construct (Origene SC118652). The polyclonal antibody ab93374—as the only one yielding reproducible results-, revealed a band at the expected size of 20 kd in western blot (Fig. 1 left panel) and a punctuate pattern in the immunofluorescence analyses (Fig. 2). When analysed for colocalisation with anti-complexIV mitochondrial subunit antibodies for mitochondrial and with anti-catalase antibodies for peroxisomal localisation, the Image J program revealed positive colocalisation for both. Remarkably, in both, western blot and immunofluorescence analyses, ab93374 reactivity was only detected in *MPV17* expression construct transfected cells (Fig. 1 left panel, Fig. 2), while endogenous MPV17 protein was not traceable. Thus the ab93374 reaction was not very sensitive, because the endogenous *MPV17* gene has been shown to be almost ubiquitously expressed at relatively high levels [25]. Furthermore, the apparent ambiguous localisation was unexpected, suggesting being potentially caused by transfection and overexpression artifacts. Therefore, mouse monoclonal antibodies raised against a recombinant Glutathion-S-Transferase (GST)-human MPV17 fusion protein, in which GST was fused to the N-terminus of MPV17 [26], were studied. Two of them were reactive to the bacterially expressed fusion protein but not to GST alone [26].

**Fig. 3:**
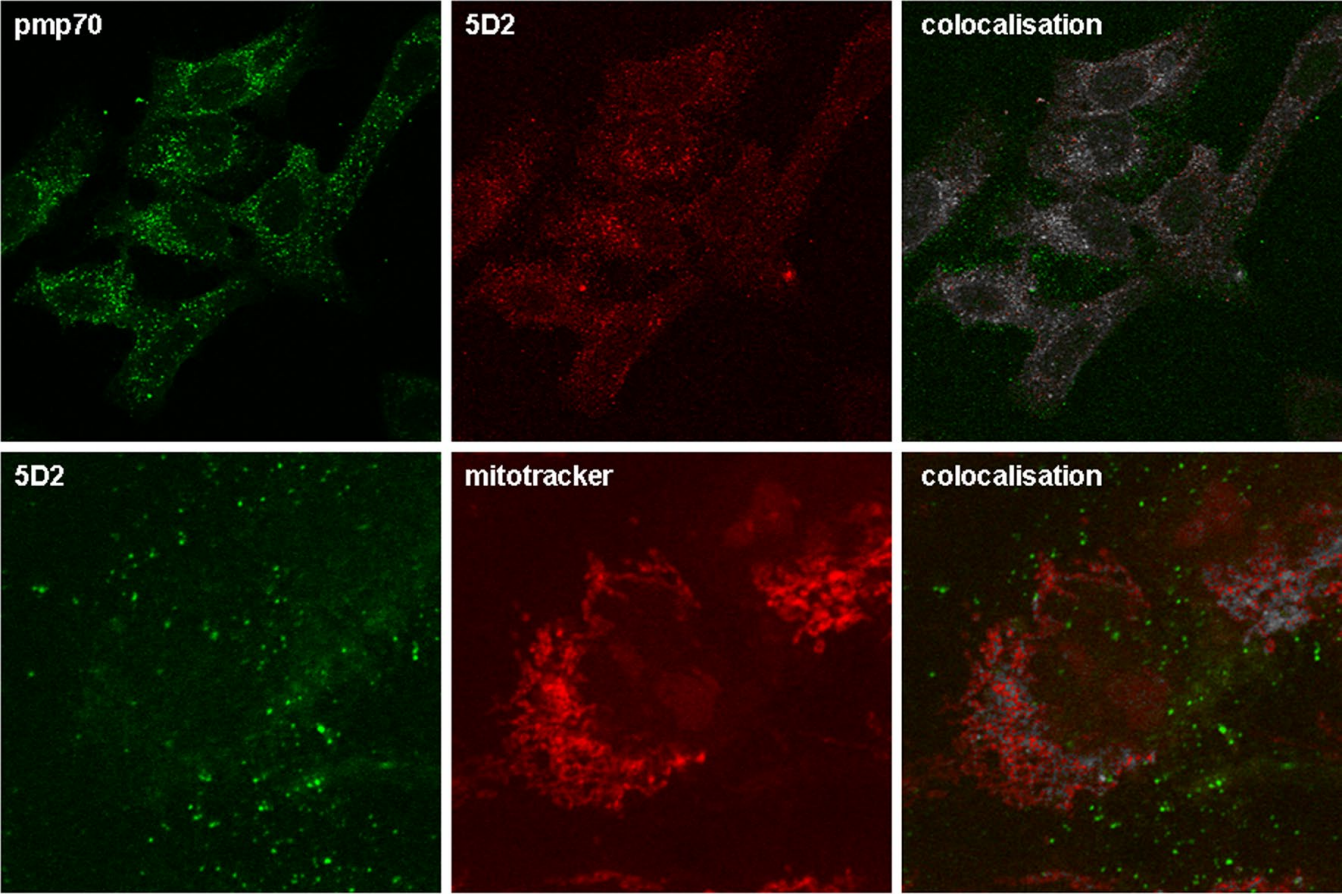
In U2OS cells, 5D2 antibody detects a punctate pattern colocalising with a peroxisomal (PMP70) but not with a mitochondrial (MitoTracker) marker. *Top* Rabbit anti PMP70 antibody (*decorated green*) was a gift from W. Just, Heidelberg. Secondary antibodies: Alexa Fluor 555 anti-mouse and Alexa Fluor 488 anti-rabbit. *Bottom* Mitotracker 7510 (Invitrogen) was used as recommended by the supplier. Secondary antibody: Alexa 488 anti-mouse. IF method and microscopy according to Kinkley et al. [10] using a Zeiss LSM confocal microscope. Mathematical colocalisation analysis was performed using the Fiji software of Image J and is illustrated by *white dots*

These two, 6F5 and 5D2, were tested on Western blots of human U2OS cell extracts (Fig. 1, central panel). Both showed reactivity against a protein of the expected size of approximately 20 kD. 6F5, however, recognised a number of additional bands evidencing cross-reactivity with other proteins. 5D2, by contrast, only detected a single band at 20 kD, the expected size. To investigate the specificity of these signals *MPV17* negative cells were necessary. For lack of such human cells from MDDS patients we tested these antibodies on extracts of mouse embryo fibroblasts (MEF) derived from *MPV17* +/+ and *MPV17* −/− cells from *MPV17* knockout mice [26], respectively (Fig. 3, right panel). It revealed that the 20 kD band recognised by 6F5 was indeed the only one missing in *MPV17* −/− cells, indicating that this band actually represented the MPV17 protein. 5D2, in contrast to the human cells, only showed a very weak signal on the mouse cells but this was again specific for the *MPV17* positive genotype. Taken together, 6F5 recognised an epitope on the MPV17 protein, which this protein shares with a number of other proteins. On the other hand, 5D2 apparently displays exclusive anti-MPV17 reactivity, strong in human U2OS cells but very weak in murine cells *MPV17* positive and absent in *MPV17* negative cells. This may indicate, that 5D2 is specific for a human specific epitope on the MPV17 protein, which is 92 % identical between mouse and man [9]. Because the anti-human antibody was raised in mice, there might be a preference for such epitopes in the immune response.

When tested in immunofluorescence studies on U2OS cells, 6F5 did not display specific reactivity (data not shown), while 5D2 generated a characteristic punctate pattern, which in the Image J analysis of confocal microscopic pictures showed partial colocalisation with the peroxisomal marker PMP70 (polyclonal rabbit PMP70 antibody, gift from W. Just, University of Heidelberg), (Fig. 3 top). However, the pattern did not coincide with mitochondrial staining as displayed by MitoTracker Red (MP07510, Invitrogen) (Fig. 3 bottom). Thus, the staining pattern was not exclusively peroxisomal and not mitochondrial, as had been reported for MPV17 localisation in earlier studies, respectively [21, 26].

**Fig. 4:**
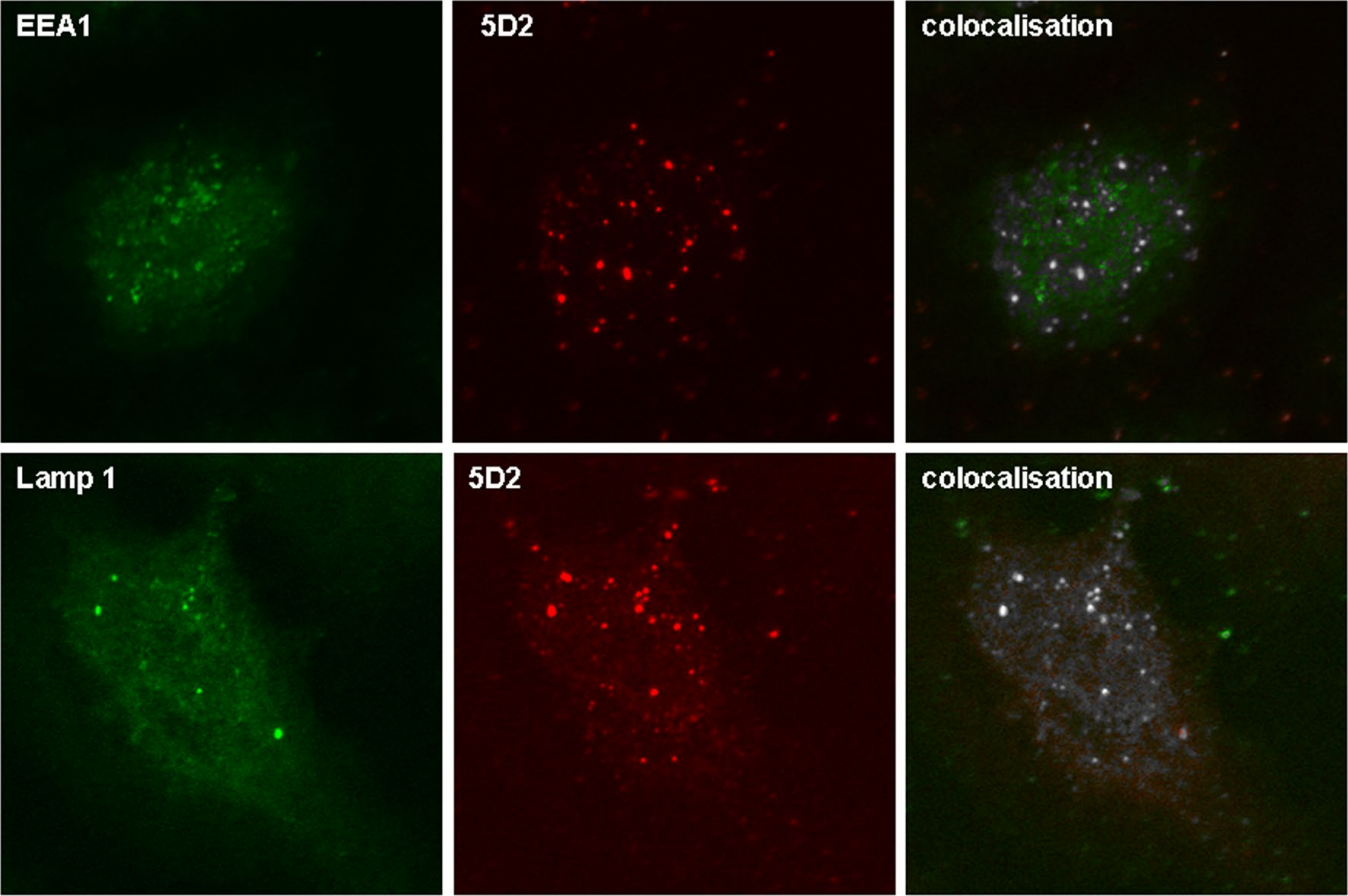
5D2 colocalisation with a marker of early endosomes (EEA1) and lysosomes (LAMP1) in U2OS cells. *Top* Partial colocalisation of 5D2 with EEA1 (rabbit anti-human). *Bottom* colocalisation of 5D2 with LAMP1 in U2OS cells. The IF method, secondary antibodies, and Image J analysis were used as described in the legend of Fig. 3

**Fig. 5:**
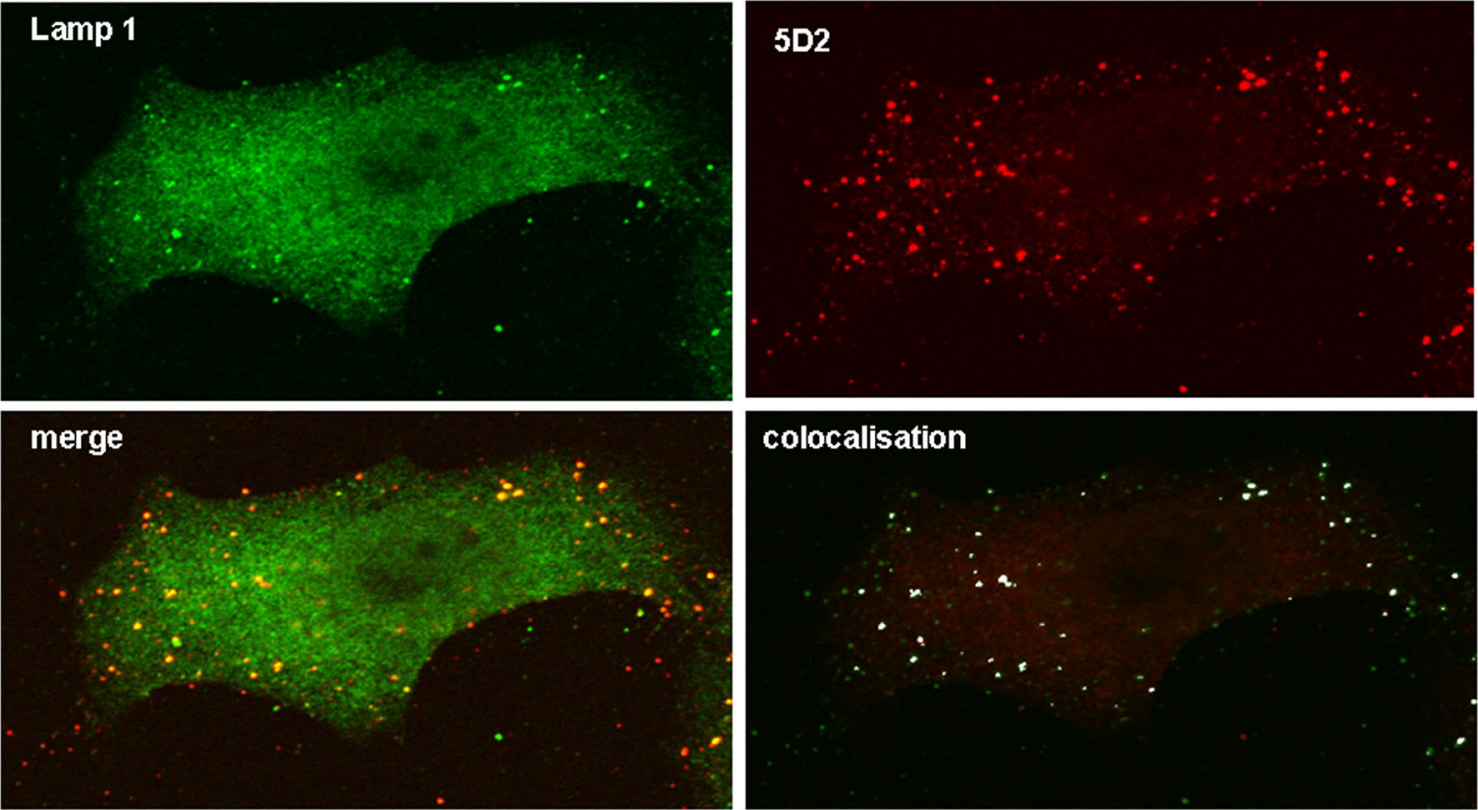
Colocalisation of 5D2 and LAMP1 mediated immunofluorescence in a U2OS single cell. IF and analysis of 5D2 and Colocalisation of 5D2 with LAMP1 in U2OS cells. The IF method, secondary antibodies, and Image J analysis were used as described in the legend of Fig. 3. In addition a merge of the two immunofluorescence pictures is depicted

In search of other co-localising structures, experiments were performed with markers for other organels. As depicted in Fig. 4 we found partial colocalisation to a marker of the early endosomal compartment, EEA1 (Fig. 4 top), while there was no apparent co-localisation with RAB7, a marker of the late endosomal compartment (data not shown). In addition, clear colocalision was detected with Cathepsin D (not shown) and LAMP 1 (Fig. 4 bottom). Figure 5 shows a single cell analysis of LAMP1—5D2 costaining, in which in addition the optical overlay colocalisation was added to the mathematical colocalisation analysis by the Fiji software of the ImageJ program package. Thus, 5D2 recognizes a structure that is present in endosomes and lysosomes in addition to peroxisomes. This in marked contrast to earlier localisations of MPV17 with different antibodies.

### Conclusions

In this work we have characterised two monoclonal antibodies raised against a bacterially expressed N-terminal GST fused to the human MPV17 protein. These antibodies, specific for the MPV17 part of the fusion protein recognise a band of the expected size in human U2OS cells. While 6F5 cross reacts with other proteins 5D2 appears to be monospecific. 5D2 creates a punctate pattern in immunofluorescence studies partially colocalising with peroxisomal, early endosomal, and lysosomal markers but not with mitochondria. 5D2 does bind only weakly but specifically to the murine MPV17 homolog (Fig. 1), suggesting that the mouse monoclonal might bind to an epitope where murine and human MPV17 diverge. This could be the c-terminus of the molecule, which is the region of strongest divergence. In line with this idea is the fact that the c-terminus of the MPV17 molecule was exposed in the bacterial n-terminal fusion to GST antigen used for immunisation. Moreover, MPV17 molecules fused at the c-terminus to other proteins cannot be detected by 5D2 (data not shown).

The commercial anti-human MPV17 antibody used in our first approach was a polyclonal rabbit antibody raised against GST-MPV17 fusion protein (abcam ab 93374) and has produced erroneous or ambiguous results (Fig. 2). The rabbit polyclonal antibody from Proteintech used by [21] leading to mitochondrial localisation was raised against the identical GST-MPV17 fusion and might have been error—prone as well. Furthermore, in addition to possible transfection artifacts, MPV17 and related proteins such as PXMP22 may localise erroneously, when they are fused to detection tags (unpublished and [3].

*MPV17* deficiencies in humans can cause fatal liver disease mediated by mitochondrial DNA depletion in liver cells [21]. However, primary human liver cells were not available for our studies. We therefore had to perform this study on unrelated human tumour cells, and it is possible, that the MPV17 protein might localise differently in different cell types. Yet, preliminary studies on human primary skin fibroblasts show MPV17 colocalisation with LAMP1 as well (unpublished results) and thus corroborate the data on U2OS cells presented above. Thus, our data raise the question of how the mitochondrial MDDS phenotype is generated if the *MPV17* gene product is found mainly in other organelles. It has been shown that MPV17 protein forms a membrane channel with a diameter allowing low molecular weight molecules to pass [1] but the role of the channel is still elusive, particularly considering the different phenotypes that mutations of it can cause in different species [13]. We look forward to provide the antibodies described here to approach these open questions.
